# Associations between Forkhead Box O1 (FoxO1) Expression and Indicators of Hepatic Glucose Production in Transition Dairy Cows Supplemented with Dietary Nicotinic Acid

**DOI:** 10.1371/journal.pone.0146670

**Published:** 2016-01-22

**Authors:** Asako Kinoshita, Lena Locher, Reka Tienken, Ulrich Meyer, Sven Dänicke, Jürgen Rehage, Korinna Huber

**Affiliations:** 1 Department of Physiology, University of Veterinary Medicine Hannover, Foundation, Hannover, Lower Saxony, Germany; 2 Clinic for Ruminants with Ambulatory and Herd Health Services at the Center of Veterinary Clinical Medicine, Ludwig-Maximilians-University Munich, Munich, Bavaria, Germany; 3 Institute of Animal Nutrition, Federal Research Institute for Animal Health, Friedrich-Loeffler-Institute, Braunschweig, Lower Saxony, Germany; 4 Clinic for Cattle, University of Veterinary Medicine Hannover, Foundation, Hannover, Lower Saxony, Germany; University of Alabama at Birmingham, UNITED STATES

## Abstract

Forkhead box protein O1 (FoxO1) is a transcription factor which promotes hepatic glucose production (HGP) by up-regulating the transcription of gluconeogenic enzymes in monogastric species. The activity of FoxO1 is inhibited by insulin-induced phosphorylation. The aims of the present study were to find associations between FoxO1 expression and variables associated with HGP as affected by feeding regimen in dairy cows during the transition period. Twenty one healthy German Holstein cows were allocated to four groups (LC-CON, HC-CON, LC-NA with 5 cows/group and HC-NA with 6 cows/group, respectively). Cows received 0 (LC-CON and HC-CON) or 24 (LC-NA and HC-NA) g/d nicotinic acid with high (HC) or low (LC) concentrate proportion from -42 days (-41.8 + 4.8; mean + standard deviation) relative to expected calving date (d-42) to d24. Liver biopsy was taken at d-42, 1, 21, and 100. The total protein expression of FoxO1 (tFoxO1) and the extent of phosphorylation of FoxO1 at serine 256 (pFoxO1) were analysed semiquantitatively by Western Blotting. The expression of hepatic mRNA of FoxO1 and seven genes associated with HGP was measured by real-time RT-PCR. Mixed model and Pearson’s correlation were used for statistical evaluation with the level of significance at P<0.05. No dietary effect was observed either on feed intake, energy balance, or on the concentration of blood metabolites. Neither time nor diet affected the expression of FoxO1 total protein and mRNA. A NA × concentrate interaction was found in pFoxO1. However, no corresponding dietary effect was found in the mRNA expression of investigated genes. Different patterns of correlations between FoxO1-related variables and investigated indicators for HGP were found at d21 and 100. The results indicated that the regulation of HGP did not take place on the levels of mRNA and protein expression and the phosphorylation of FoxO1 in dairy cows in early lactation.

## Introduction

Forkhead box protein O1 (FoxO1) is a member of a transcription factor protein family FoxO. The protein family FoxO shares a common DNA-binding domain, which consists of 110 amino acids named forkhead box- or winged helix domain. In laboratory animals, it has been shown that FoxO1 plays a central role in cellular proliferation, differentiation, DNA damage repair response, and in metabolic regulation [1]. The activity of FoxO1 is regulated by external stimuli, such as insulin, insulin-like growth factor (IGF-1), nutrients, and cytokines. The regulation takes place by controlling the levels of FoxO1 mRNA or protein, posttranslational modifications, and interactions with other proteins [2]. The FoxO1 protein is one of the main targets of insulin/IGF-1 signaling pathway. Insulin phosphorylated FoxO1 through PI3K-Akt signaling. The phosphorylation of FoxO1 by insulin inhibited the binding of FoxO1 to the target DNA and induced a rapid translocation of FoxO1 protein from nucleus to cytoplasm. This led to the deactivation of FoxO1 [2]. In the liver of monogastric species, FoxO1 is a main regulator of glucose production. In its active form, the FoxO1 protein bound to the FoxO-responsive elements in the promoter region of glucose-6-phosphatase (G6P) and cytosolic phosphoenolpyruvate carboxykinase (PCK1) and promoted the transcription of these genes, this leading to an increased hepatic glucose production [1,3,4]. In cattle, studies showed that FoxO1 appeared to be associated with enhanced gluconeogenesis in the liver in underfed cows [5]. However, the exact role of FoxO1 in hepatic gluconeogenesis and its association with insulin sensitivity in dairy cows were not investigated on the translational and post-translational levels.

Dairy cows with high milk yield undergo energy shortage in the first 100 days after calving. This is because the increasing energy demand for milk synthesis cannot be covered by dietary energy intake. Cows in this period had lower insulin concentration and had lower tissue responsiveness to insulin [6]. In dairy cows more than 50% of the glucose demand was covered by hepatic glucose production (HGP) [7]. In vivo studies have shown that the glucose output from liver increased largely due to the onset of lactation [7]. Increases in the amount of mRNA of gluconeogenic enzymes caused by the onset of lactation have also been observed [8–10]. The hepatic gluconeogenesis in ruminants appeared to be insensitive to inhibitory effects of insulin [6, 11]. However, little is known about the factors modulating insulin insensitivity of HGP in dairy cows.

Nicotinic acid (NA) has been reported to affect energy metabolism in dairy cows through several mechanisms. Supplementation of NA can modify the ruminal fermentation and increase the proportion of propionate [12,13]. After being absorbed, NA was an important precursor for NAD synthesis [13]. Studies in vitro [14] and in vivo [15,16] stated that NA had an inhibitory effect on lipolysis in dairy cows. This effect was caused by activating the nicotinic acid receptor [14,17]. In addition, recent studies demonstrated that NA induced transcriptional, translational and post-translational modifications in the skeletal muscle in sheep [18] and insulin sensitive tissues (liver, muscle and adipose tissues) in rats [19]. The study in rats indicated that a part of effects of NA on expression of genes could be associated with NA-induced changes in post-translational modification of FoxO1 [19].

We assumed that FoxO1 promotes transcription of genes associated with HGP, and that the phosphorylation of FoxO1 by insulin decreases the transcription of genes associated with HGP in dairy cattle during the transition period. Based on these assumptions, we hypothesized that there are cross-linked associations between the expression of FoxO1, the extent of its phosphorylation, the metabolic status, serum insulin concentration and the expression of hepatic mRNA of genes associated with HGP. To test the hypothesis, the expression and the extent of phosphorylation of hepatic FoxO1 in dairy cows were monitored during the transition period. Moreover, correlations for FoxO1 with dry matter intake (DMI), serum insulin and non-esterified fatty acid (NEFA) concentration, as well as the hepatic mRNA expression of genes associated with HGP were examined. Cows were divided into four experimental groups and underwent the feeding strategies to induce mild or severe negative energy balance after calving with or without dietary NA supplementation.

## Materials and Methods

### Animals and Feeding

The experiment was conducted according to the European Community regulations concerning the protection of experimental animals and the guidelines of the Lower Saxony State Office for Consumer Protection and Food Safety (LAVES), Oldenburg, Germany. The approval of Institutional Animal Care and Use Committee (IACUC) was not obtained, because all the necessary assessments regarding the protection of animal welfare and ethics during the animal experiment was conducted by LAVES. The animals used in this study were a subset of a more comprehensive study with a larger number of animals [20,21]. The feeding strategy of the trial and the results of analysis of performance data were described in detail elsewhere [20,21]. In brief, twenty-one pluriparous German Holstein cows were allocated to four groups (LC-CON, HC-CON, LC-NA with 5 cows/group and HC-NA with 6 cows/group). Two of them (LC-CON and LC-NA) were fed a diet containing low concentrate proportion (30% dry matter basis) from about 42 days before calving (-41.8 + 4.8; mean + standard deviation) to calving. After calving, the proportion of concentrate was increased to 50% within 16 days. The other two groups (HC-CON and HC-NA) received a diet containing high concentrate proportion (60% DM basis) from about 42 days before calving to calving. After calving, the proportion of the concentrates was set at 30% and increased to 50% within 24 days. The cows in LC-NA and HC-NA received 1 kg/day of pelleted concentrate including non-rumen protected NA (Mianyang Vanetta Pharmaceutical Technology Co., Ltd, Sichuan, China) at 24 g/day from about 42 days before calving to 24 days after calving, while the cows in HC-CON and LC-CON received 1 kg of a control concentrate. Roughage for *ad libitum* intake and concentrate for the intake of the adjusted amount were offered via a computerized self-feeding station (Insentec B.V., Marknese, The Netherlands) separately, where the daily individual DMI was recorded. All the cows were healthy before, during and after the feeding experiment.

### Sampling

Blood samples were collected from the *vena jugularis externa* at 7.30 a.m. on days on which cows were expected to calve, at d-42, d-14, d-7, and after calving at d1, d7, d14, d21, d42, d63, and d100. The blood samples were centrifuged at 2000 *g* for 15 min at 15°C. The obtained blood serum was stored at -80°C until analysis. Liver biopsies were taken transcutaneously under sonographic control (SSA 370 A, Toshiba, Tokyo, Japan) at d-42, d1, d21, and d100 from the right 10^th^ or 11^th^ intercostal space (Bard Magnum, Tru-Cut 12G needle, Bard Biopsy Systems, USA). At the insertion site of the biopsy-needle the skin was clipped, washed, degreased with medical alcohol and disinfected with iodine (Vetisept, Dr. E. Graeub AG, Bern, Switzerland) and the abdominal wall was infiltrated with 5 mL procaine (Procaine 2%, Selectavet, Dr. Otto Fischer GmbH, Weyarm-Holzolling, Germany) for local anesthesia. Cows received ketoprofen (3 mg/kg body weight IV; Romfen PR, Merial GmbH, Halbergmoosm, Germany) as analgesia and ceftiofur (6.6 mg/kg body weight SC; Naxcel, Zoetis Belgium) as an antibiotic treatment after biopsies were taken. Liver biopsy samples were shock-frozen in liquid nitrogen and stored at -80°C until analysis.

### Analysis of blood samples

Serum insulin concentration was measured by radioimmunoassay (IM3210, Immunotech, Beckman Coulter Inc., Brea, CA, USA). The intra- and inter- assay variance coefficient was 7.6% and 10.7%, respectively for the radioimmunoassay for insulin concentration. Serum NEFA concentration used for correlation analysis was determined by enzymatic reactions, using spectrometric detection (Eurolyser CCA 180 Vet system, Eurolyser Diagnostica GmbH, Salzburg, Austria).

### Western blot of hepatic tissue for total and phosphorylated FoxO1 protein expression

Liver samples (30–60 mg) were homogenized in 800 μL lysis buffer [in m*M*: 50 HEPES, pH 7.4; 0.1% Triton X-100 (vol/vol); 4 ethylene glycol-bis(2-amino-ethylether)-N,N,N´,N´-tetraacetic acid (EGTA); 10 EDTA; 100 beta-glycerophosphate; 15 tetrasodium pyrophosphate; 5 sodium orthovanadate; 2.5 sodium fluoride] containing protease inhibitors (cOmplete, Mini, F. Hoffmann-La Roche Ltd., Basel, Switzerland) and phosphatase inhibitors (PhosStop; F. Hoffmann-La Roche Ltd.) with ceramic beads (Matrix-Green, MP Biomedicals, Santa Ana, USA) using the BIO101 Thermo Savant FastPrep FP120 Homogenizer (Qbiogene Inc., Carlsbad, USA). The tissue homogenates were centrifuged at 9000 *g* for 5 min at 4°C. The supernatant was aliquoted and shock frozen in liquid nitrogen and stored at -80°C until further analysis. Protein concentrations of the samples were determined by Bradford assay (Bradford Reagent, SERVA, Heidelberg, Germany) according to the manufacturer’s instructions. The homogenates containing 30 μg of protein were diluted in Laemmli buffer [22] [50 m*M* Tris-HCl, 10% glycerol (vol/vol), 5% SDS (wt/vol), bromphenol, and 2% mercaptoethanol (vol/vol)] in a total volume of 20 μL and were heat-denatured at 95°C for 5 min and then applied to SDS-PAGE using precast polyacrylamide gels (7.5% Mini-PROTEIN® TGX^TM^, Bio-Rad Laboratories, Inc., Hercules, USA) at 2000 V for 34 min. Protein was transferred to nitrocellulose membrane with 0.2 μm pore (Bio-Rad Laboratories, Inc.) by means of the semi-dry blotting system (Trans-Blot Turbo system, Bio-Rad Laboratories, Inc.) at 25 V for 30 min.

Membranes were treated with blocking buffer containing 10% fat-free milk-Tris -buffered saline (TBS) (50 m*M* Tris-hydrochloride, pH 7.6, 150 m*M* sodium chloride) + 1% Tween 20 (vol/vol) at room temperature for 60 min, and afterwards three washing cycles with TBST (TBS + 1% Tween 20) of 5 min each. Membrane was incubated with primary antibodies diluted at 1:1000 for total FoxO1 protein (tFoxO1) (rabbit anti-FoxO1 polyclonal antibody, bs9439R, Bioss Inc., Massachusetts, USA) or 1:200 for phosphorylated FoxO1 protein at serine 256 (pFoxO1) (rabbit anti-FoxO1 (Ser256) polyclonal antibody, bs-3142R, Bioss Inc.), as well as 1:10000 for beta-actin (mouse anti-beta-actin monoclonal antibody, A5441, Sigma-Aldrich, Inc., Missouri, USA) in 5% skimmed milk-TBST over night at 4°C, followed by three wash cycles in TBST of 5 min and incubation with secondary antibodies diluted at 1:20000 for tFoxO1 and pFoxO1 (goat anti-rabbit IgG-peroxidase antibody, A545, Sigma-Aldrich, Inc.) as well as at 1:10000 for beta-actin (goat anti-mouse IgG-peroxidase antibody, A2304, Sigma-Aldrich, Inc.) in 5% skimmed milk-TBST at room temperature for one hour. Membranes were then washed three times for 5 min with TBST and once for 10 min with TBS and incubated with LumiGLO substrate (Kirkegaard & Perry Laboratories, Inc., Gaithersburg, USA). Detecting and recording chemiluminescence signals of membranes were performed using a Molecular Imager ChemiDoc XRS+ System (Bio-Rad Laboratories, Inc.).

Signals for tFoxO1 and pFoxO1 were detected at 70 kD and signals for beta-actin were detected at 45 kD (S1 and S2 Figs). The specificity of the used antibodies was confirmed by the molecular weight of signals and by the manufacturer’s statement regarding the reactivity of the antibodies with bovine proteins in western blotting. The antibodies used in this study were raised against the human protein for FoxO1 and phosphorylated FoxO1 at serine 256. However, these antibodies were also able to detect the signals of bovine proteins at the targeted molecular weight as stated by the manufacturer. The amino acids sequences of bovine FoxO1 is up to 87.9% identical to those of human FoxO1. Accordingly, the amino acid sequence around serine 256 (Arg151-Arg267) in human FoxO1 is 100% identical to the amino acids sequence in the corresponding region in bovine FoxO1. Therefore, the antibodies used in this study detected the proteins that are equivalent to human tFoxO1 and pFoxO1 specifically.

The analysis was repeated twice for each cow. Data were processed and analyzed by densitometry using Image Lab software (Ver. 4.1, Bio-rad Bio-Rad Laboratories, Inc.). The normalizations for the equalities of loading for each signal as well as for the equalities of treatments for each membrane were performed using beta-actin and inter-membrane control samples, respectively.

### RNA isolation, RNA quality control, and reverse transcription

Total RNA was isolated using a commercial kit (RNeasy® Mini Kit, Qiagen, Hilden, Germany) according to the manufacturer’s instruction. Liver samples (about 30 mg) were homogenized in 600 μL homogenizing buffer containing guanidine thiocyanate and beta-mercaptoethanol as described above. After centrifugation for 3 min at full speed, 400 μL of the supernatant was mixed with 400 μL 50% ethanol and transferred to the spin column for RNA binding. The spin column was washed and incubated with DNase I (DNase-Free DNase Set, Qiagen, Hilden, Germany) for 15 min at room temperature to digest the possible genomic DNA contamination. After three cycles of washing, the column was centrifuged for 1 min for drying. RNA was eluted in a total volume of 65 μl of nuclease-free water. The concentration and purity of isolated RNA was determined by measuring absorbance OD 230, 260, and 280 nm (Biophotometer, Eppendorf AG, Hamburg, Germany) of samples diluted at 1:15 in 2 m*M* disodium hydrogen phosphate (Na_2_HPO_4_) (pH 8.0). The integrity of isolated RNA was controlled using RNA integrity number measured with Agilent 2100 Bioanalyzer and a commercial kit (RNA Nano Chips, Agilent Technologies, Santa Clara, USA) according to the manufacturer’s instructions. The RNA concentration of samples, A260/A280 ratio, A260/A230 ratio as well as RIN of samples (mean ± standard deviation) were 420.4 ± 109 (range 209–660) ng/μL, 2.2 ± 0.0 (range 2.1–2.3), 2.0 ± 0.3 (range 0.54–2.5), and 8.2 ± 0.3 (range 7–8.6), respectively. The lower A260/A230 ratio under 2.0, an indicator for the contamination of chloroform, which has inhibitory effects on downstream analysis, was observed in 32 samples. These samples were further analyzed together with others, because chloroform was not used in the present protocol, and no negative effects of lower A260/230 ratio on downstream analysis had been observed in preliminary studies. Reverse transcription of isolated RNA was performed using a commercial kit (iScript cDNA Synthesis Kit, Biorad Laboratories, Inc., Hercules, USA) according to the manufacturer’s instructions. One μg of total RNA was mixed with the reaction mix, reverse transcriptase and nuclease-free water with a final volume of 20 μl and incubated for 5 min at 25°C for stabilization, followed by incubation for 30 min at 42°C for reverse transcription and for 5 min at 85°C for denaturation. Transcribed cDNA samples were cooled immediately to 4°C, diluted at 1:20 with nuclease-free water, aliquoted for single use, and stored at -20°C for further analysis. No template controls and no reverse transcriptase controls were included in the assay.

### Quantitative real-time PCR

Quantitative real-time PCR assay was performed using the CFX96 Touch Real-Time PCR Detection System (Biorad Laboratories, Inc., Hercules, USA) and commercial PCR master mix (SsoAdvanced™ Universal SYBR® Green Supermix, Biorad Laboratories, Inc., Hercules, USA) according to the manufacturer’s instruction. The reaction mix consisted of PCR master mix including polymerase, dNTPs, MgCl_2_, and SYBRGreen I dye, reverse and forward primer (500 n*M* for each), cDNA samples (0.1 μL of undiluted samples corresponding to 5 ng of RNA) and nuclease-free water with a final volume of 20 μL. The assays were performed in triplicate. The thermal cycling protocol was 30 sec at 95°C for polymerase activation, 40 cycles of amplification steps consisting of 10 sec at 95°C and 15 sec at 60°C, and melt-curve analysis, from 65°C to 95°C, 5 sec at 0.5°C increments. Each assay contained no template controls and standards (mixture of all the cDNA samples) for the standard curve (5 dilution series corresponding to 1.25–20 ng RNA) as well as for inter-plate controls. The investigated genes were FoxO1 (FoxO1), propionyl CoA carboxylase A (PCCA), pyruvate carboxylase (PC), PCK1, G6P, glucose transporter 2 (SLC2A2, solute carrier family 2 (facilitated glucose transporter), member 2), insulin receptor isoform A and B (IRA and IRB), and glycogen phosphorylase, liver form (PYGL). Primers were selected using the online tool for primer design “Primer 3” [23] by setting the selection criteria including CG contents at 50%, primer length at 20 base pair, target product length at 150–300 base pair, annealing temperature at 60°C, with primers or target products spanning a large intron. Specificity of the PCR products were tested by melt-curve analysis, agarose gel electrophoresis, and sequencing (Eurofins Genomics GmbH, Ebersberg, Germany), followed by BLAST analysis (NCBI, Bos taurus Nucleotide BLAST) [24] confirming a single melting peak and a single band for each target amplicon (S3 Fig). All the sequences of PCR products were found specifically in the target sequences (S1 Table). Investigated genes, primer sequence, and PCR conditions are shown in Table 1. Reference genes were selected by analyzing stability of expression of seven candidate genes (ribosomal protein L19: RPL19, ribosomal protein L32: RPL32, ribosomal protein S9: RPS9, ubiquitously expressed transcript: UXT, ribosomal protein S15: RPS15, RNA binding motif, single strand interacting protein 2: RBMS2, mitochondrial ribosomal protein L39: MRPL39) in 28 samples from all the experimental groups and sampling days. The geNorm analysis performed using qBase Plus (Biogazelle NV, Zwijnaarde, Belgium) revealed that RPL19 and RPL32 were expressed most stably for all the samples. The geNorm V was 0.085 (calculated using 28 samples) and because it was under the threshold of 0.15 [25], the third reference gene was not measured. The coefficient of variation of the normalized reference gene expression levels and the geNorm stability M-value were 0.096 and 0.272, respectively in the expression of two selected reference genes in all 84 samples. The further results of qPCR assay were analyzed also using qBase Plus. Normalized relative quantities of RNA for target genes were calculated from the threshold cycles (Cq) at relative fluorescence units of 50 using the delta-delta-Ct method-based method modified for the use of multiple reference genes for normalization [26] by setting the mean value of all samples at 1 for each gene. The amplification efficiency was set to 2 because the estimated amplification efficiency from the standard curve did not differ much from 2 within the investigated dynamic range (1.25–20 ng RNA) for all the measured genes (Table 1). In raw data quality control, the Cq values with differences greater than 0.5 cycles to other ones were discarded.

**Table 1:** Characteristic of primers and the real-time PCR conditions.

Symbol	Primer sequences (5´– 3´)^1^	Accession number^1^	Size^2^	Tm^2^	E^3^	slope (r^2^)^3^	Mean Cq^4^
FoxO1^5^	F-CGCAGATTTACGAGTGGATGG	XM_583090	189	85	2.1	-3.07	24.6
	R-CACTCTTGCCTCCCTCTGG					(0.98)	
G6P	F-GATAAAGCAGTTCCCGGTCA	BC114011	199	85	2.1	-3.14	19.5
	R-GCAGACATTCAGTTGCACGA					(0.99)	
SLC2A2	F-CGAAATTGGGACCATCTCAC	BC149324	207	84	2.0	-3.29	21.7
	R-TGCCCAGGATAAAGTCAAGG					(1.00)	
IRA^6^	F-TCCTCAAGGAGCTGGAGGAGT	AJ320235	89	81.5	2.0	-3.32	26.7
	R-TTTCCTCGAAGGCCTGGGGAT					(0.99)	
IRB^6^	F-TCCTCAAGGAGCTGGAGGAGT	AJ320235	110	83	2.1	-3.20	24.5
	R-TAGCGTCCTCGGCAACAGG					(0.99)	
PC	F-CCAGAAAGTGGTGGAGATCG	BC114135	298	90.5	2.1	-3.13	21.9
	R-GTTGATGCGGATGTTCTCCT					(0.99)	
PCCA	F-AACCGCAGAAGCTGCTACAT	BC123876	181	84.5	2.0	-3.26	23.3
	R-CACTGTGCCGAGAAACTGAA					(0.99)	
PCK1	F-GCCTGACCAAGTCCACATCT	BC112664	198	86.5	2.0	-3.33	18.7
	R-ATGGGCACCGTATCTCTTTG					(0.97)	
PYGL	F-CAACGTGAAGCAGGAGAACA	BC120097	195	85	2.1	-3.17	20.2
	R-GGCACGAATAGCTTCTTTGG					(1.00)	
RPL19	F-GGTACTGCCAATGCTCGAAT	BC102223	200	84.5	2.0	-3.23	18.4
	R-TTGTCTGCCTTCAGCTTGTG					(0.99)	
RPL32	F-CCTCGTGAAGCCTAAGATCG	BC102748	229	86	2.0	-3.22	18.4
	R-GACGTTGTGGACCAGGAACT					(0.98)	
RBMS2^5^	F-ATGGCACCACCTAGTCCAAG	BC102935	251	86	2.0	-3.26	24.1
	R-ACTGCTTTCTGTGCTGCTGA					(0.98)	
MRPL39	F-ACTGCTTTCTGTGCTGCTGA	BC122667	264	82.5	2.1	-3.13	24.5
	R-GGCACAAGAACGCCAATAAG					(0.98)	
UXT^6^	F-GGCACAAGAACGCCAATAAG	BC108205	101	81.5	1.9	-3.47	23.5
	R-GGTTGTCGCTGAGCTCTGTG					(0.99)	
MRPS15^6^	F-GCAGCTTATGAGCAAGGTCGT	BC122687	151	84	2.0	-3.24	23.5
	R-GCTCATCAGCAGATAGCGCTT					(0.97)	
RPS9	F-GGTCTGGAGGGTCAAATTCA	BC148016	171	88.5	2.0	-3.26,	19.5
	R-CCCAGGATGTAATCCAGCTT					(0.99)	

FoxO1: Forkhead box protein O1, IRA: Insulin receptor isotype A, IRB: Insulin receptor isotype B, SLC2A2: Glucose transporter 2 (solute carrier family 2 (facilitated glucose transporter), member 2), G6P: Glucose-6-phosphatase, PCK1: Cytosolic phosphoenolpyruvate carboxykinase, PCCA: Propionyl-CoA carboxylase, PYGL: Glycogen phosphorylase, RPL19: Ribosomal protein L19: Ribosomal protein L32, RPS9: Ribosomal protein S9, UXT: Ubiquitously expressed transcript, MRPS15: Mitochondrial ribosomal protein S15, RBMS2: RNA binding motif, single strand interacting protein 2, MRPL39: Mitochondrial ribosomal protein L39

^1^Forward (F-) and reverse (R-) primer sequences and NIH GenBank accession number of template sequences.

^2^Amplicon size (base pair) and melting temperature (Tm) (°C) of PCR products

^3^Amplification efficiency (E), slope and r^2^ estimated from standard curves using 5 dilution series of cDNA samples corresponding to 1.25–20 ng RNA.

^4^Mean threshold cycle (Cq) of all the measured samples at relative fluorescence unit of 50 and 0.1μl cDNA (5 ng RNA) input.

^5^Modified assay conditions were used; FoxO1:125/875 n*M* for F-/R- primer concentration and 10 sec at 95°C and 15 sec at 60°C for amplification step; RBMS2: 1 μ*M* primer concentration.

^6^Primer sequences were published by Neuvians et al. [27] for IRA and IRB, and by Bionaz et al. [28] for UXT and MRPS15.

### Statistical analyses

The statistical analyses were performed using SAS (Version 9.2, SAS Institute Inc., Cary, NC, USA). The effects of time and diets on serum insulin concentration, tFoxO, pFoxO1, and the relative quantities of mRNA of investigated genes were examined mixed model with repeated measures (PROC MIXED) [29] with time (d), dietary NA supplementation (NA) and concentrate proportion (concentrate) as fixed factors and the cow as a random factor. Restricted maximum likelihood was used for estimating means and Kenward-Roger degrees of freedom approximation was used to test fixed effects. The best fit covariant structures were decided from compound symmetry, component variance, and unstructured using AIC for each variable. The values at the first sampling day (d-42) were integrated as covariables into the mixed model. The normality of distribution of residues was checked using the Shapiro-Wilk test (PROC UNIVARIATE). Pearson’s correlation analysis (PROC CORR) was performed for the correlations for tFoxO1, pFoxO1, mRNA expression of FoxO1, and for the ratio of pFoxO1/tFoxO1 with all the other variables at each sampling day. The serum NEFA concentration, DMI, and pFoxO1/tFoxO1 were included in the correlation analysis only because the data had already been published (NEFA and DMI) [20,21] or no significant effect of fixed factors was found (pFoxO1/tFoxO1). Levels of significance and of relevant trend were set at P < 0.05 and 0.1, respectively in the factorial analysis. In the correlation analysis, the level of significance was set at P ≤ 0.01.

## Results

### Serum insulin concentration

**Fig 1:**
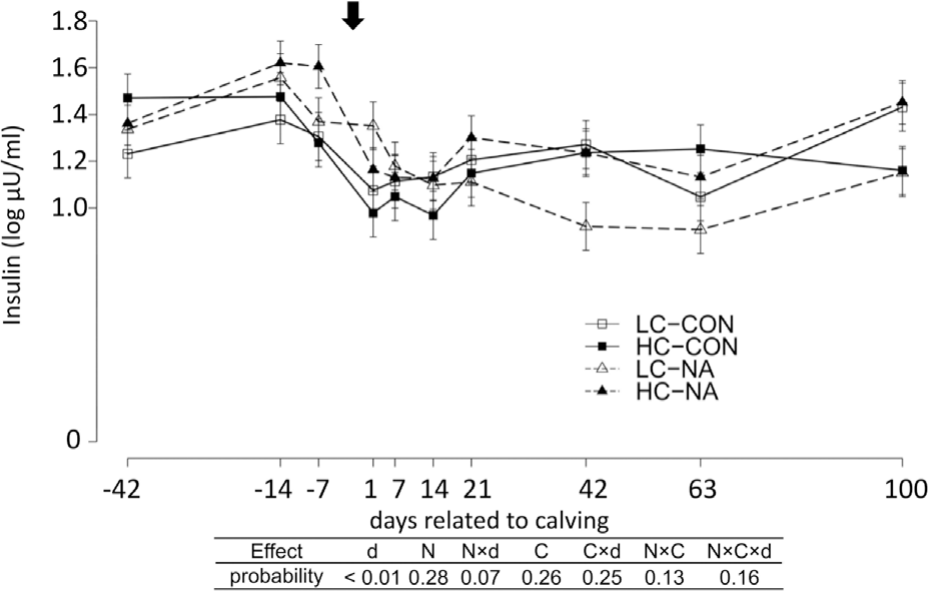
Serum insulin concentration (Log10 Insulin μU/ml). Data are shown in least squares means (LSM) ± standard errors of each experimental group and sampling day. The arrow above indicate the day of calving. The results of type 3 test for the effects of time and diets are shown in the table under the diagrams. LC-CON, HC-CON, LC-NA, HC-NA: “CON or NA”: dietary supplement of nicotinic acid (0 or 24 g/d) from d-42 to d24, “LC or HC”: 30 or 60% of concentrate proportion in the diet from d-42 to d0, increase in concentrate proportion in the diet after calving from 30 to 50% within 16 or 24 days. d: Days related to calving, N: Nicotinic acid, C: Concentrate proportion in the diet, N×d, C×d, N×C, N×C×d: interaction effect of d, N, C.

Serum insulin concentrations were significantly affected by time (Fig 1). The serum insulin concentration increased from d-42 to d-7. At d1, the serum insulin concentration decreased and maintained the lowest levels in the whole experimental period until d14. The insulin level recovered gradually until d100 to the level at d-42.

### Total protein expression (tFoxO1), extent of phosphorylation at serine 256 (pFoxO1) and mRNA expression of FoxO1

**Fig 2:**
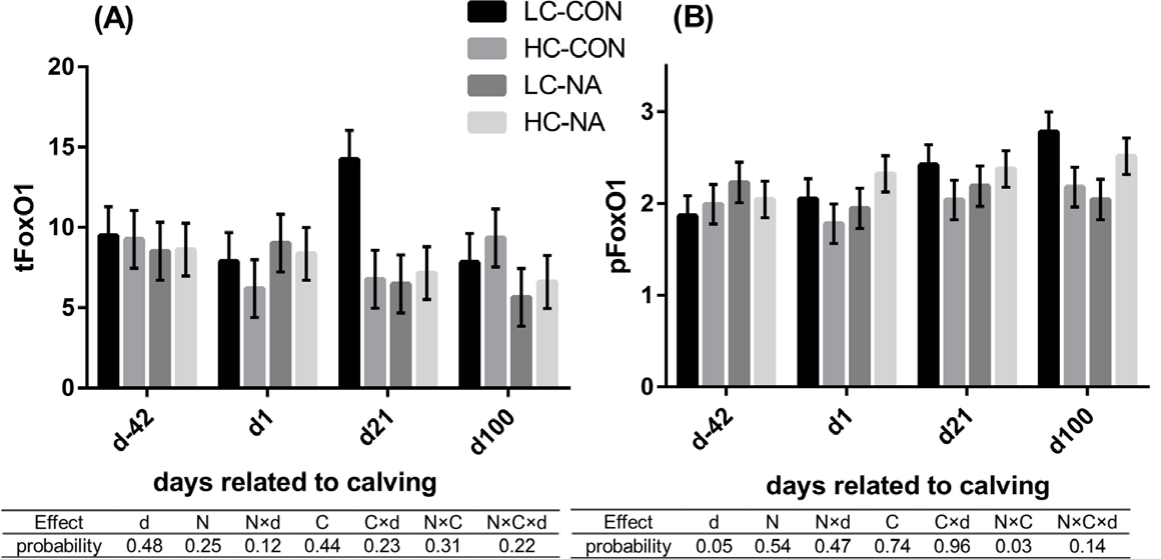
FoxO1 total protein expression (tFoxO1) (A) and the extent of phosphorylation of FoxO1 at serine 256 (pFoxO1) (B) in liver of cows. Data are shown in least squares means with standard errors in each group and at each time point. The results of type 3 test for the effects of time and diets are shown in the table under the diagrams. LC-CON (n = 5), HC-CON (n = 5), LC-NA (n = 5), HC-NA (n = 6): “CON or NA”: Dietary supplement of nicotinic acid (0 or 24 g/d) from d-42 to d24, “LC or HC”: 30 or 60% of concentrate proportion in the diet from d-42 to d0, increase in concentrate proportion in the diet after calving from 30 to 50% within 16 or 24 days. d: Days related to calving, N: Nicotinic acid, C: Concentrate proportion in the diet, d×N, d×C, N×C, d×N×C: Interaction effects of d, N, C, tFoxO1: Total protein expression of forkhead box protein O1, pFoxO1: Extent of phosphorylation of FoxO1 at serine 256.

The results for the analysis of FoxO1 are shown in Fig 2 for tFoxO1 and pFoxO1 and in Table 2 for mRNA expression of FoxO1. **The total protein** and the **mRNA** expression of FoxO1 were neither affected by time nor by the diets. An interaction between NA and concentrate levels was observed in the **pFoxO1**. The high-concentrate diet and NA supplementation down-regulated the pFoxO1 when they affected separately as a single factor. However, the pFoxO1 in HC-NA was almost at the same level as that in LC-CON (LC-CON, HC-NA > LC-NA, HC-CON).

**Table 2:** Effects of diet on the relative quantity of mRNA of investigated genes.

		LSM				Type 3 test				
		-42	1	21	100	d	N	d×N	C	d×C
FoxO1	LC-CON	1.14	1.09	0.92	1.01	0.48	0.86	0.24	0.41	0.11
	HC-CON	0.82	0.91	0.99	1.20					
	LC-NA	1.08	1.08	1.03	1.04					
	HC-NA	0.88	1.17	1.00	0.90					
	*SEM*	*0*.*11*	*0*.*11*	*0*.*11*	*0*.*11*					
IRA	LC-CON	0.95	1.24	1.18	1.02	< 0.01	0.23	0.15	0.30	0.90
	HC-CON	0.65	1.02	0.95	0.83					
	LC-NA	0.98	1.36	1.22	0.82					
	HC-NA	0.95	1.55	1.20	0.76					
	*SEM*	*0*.*16*	*0*.*16*	*0*.*16*	*0*.*16*					
IRB	LC-CON	0.98	1.24	0.85	1.05	0.02	0.21	< 0.01	0.55	0.06
	HC-CON	0.81	1.16	1.10	1.18					
	LC-NA	0.88	0.93	1.03	1.07					
	HC-NA	0.90	0.83	1.24	1.02					
	*SEM*	*0*.*09*	*0*.*09*	*0*.*09*	*0*.*09*					
SLC2A2	LC-CON	1.06	1.15	0.68	1.28	< 0.01	0.93	< 0.01	0.88	0.04
	HC-CON	1.03	1.03	0.92	1.31					
	LC-NA	0.86	0.88	0.98	1.47					
	HC-NA	1.13	0.61	1.20	1.23					
	*SEM*	*0*.*14*	*0*.*14*	*0*.*14*	*0*.*14*					
G6P	LC-CON	1.08	0.87	0.98	1.37	< 0.01	0.14	0.10	0.87	0.14
	HC-CON	0.62	1.16	0.90	1.11					
	LC-NA	0.77	0.98	1.03	1.49					
	HC-NA	0.69	0.92	1.71	1.57					
	*SEM*	*0*.*18*	*0*.*18*	*0*.*18*	*0*.*18*					
PCK1	LC-CON	0.80	1.21	1.27	1.55	< 0.01	0.66	0.03	0.89	0.71
	HC-CON	0.60	1.03	1.22	1.65					
	LC-NA	0.38	0.98	1.61	1.42					
	HC-NA	0.83	0.82	1.74	1.24					
	*SEM*	*0*.*18*	*0*.*18*	*0*.*18*	*0*.*18*					
PCCA	LC-CON	1.06	0.85	0.81	1.06	< 0.01	0.11	0.15	0.40	0.48
	HC-CON	0.85	0.77	0.94	1.37					
	LC-NA	0.95	0.75	1.23	1.26					
	HC-NA	1.11	0.85	1.26	1.39					
	*SEM*	*0*.*13*	*0*.*13*	*0*.*13*	*0*.*13*					
PC	LC-CON	0.52	2.02	1.28	0.90	< 0.01	0.59	0.35	0.90	0.94
	HC-CON	0.51	1.91	1.40	0.88					
	LC-NA	0.44	2.27	1.41	0.82					
	HC-NA	0.46	2.42	1.54	0.69					
	*SEM*	*0*.*24*	*0*.*24*	*0*.*24*	*0*.*24*					
PYGL	LC-CON	1.06	1.01	0.86	0.96	0.14	0.69	0.16	0.14	0.05
	HC-CON	0.83	0.94	1.07	1.45					
	LC-NA	1.10	0.82	1.03	1.05					
	HC-NA	0.98	0.95	1.40	1.06					
	*SEM*	*0*.*13*	*0*.*13*	*0*.*13*	*0*.*13*					

LC-CON (n = 5), HC-CON (n = 5), LC-NA (n = 5), HC-NA (n = 6): “CON or NA”: Dietary supplement of nicotinic acid (0 or 24 g/d) from d-42 to d24, “LC or HC”: 30 or 60% of concentrate proportion in the diet from d-42 to d0, increase in concentrate proportion in the diet after calving from 30 to 50% within 16 or 24 days. d: Days related to calving, N: dietary supplement of nicotinic acid, C: Concentrate proportion in the diet, d×N, d×C: Interaction effects of d, N and C. Interaction effects of N×C, d×N×C were not significant for all the variables (not shown). LSM: Least squares means, SEM: Pooled standard error of mean, FoxO1: Forkhead box protein O1, IRA: Insulin receptor isotype A, IRB: Insulin receptor isotype B, SLC2A2: Glucose transporter 2 (Solute Carrier Family 2 (Facilitated Glucose Transporter), Member 2), G6P: Glucose-6-phosphatase, PCK1: Cytosolic phosphoenolpyruvate carboxykinase, PCCA: Propionyl-CoA carboxylase, PYGL: Glycogen phosphorylase

### Relative quantities of mRNA of genes associated with hepatic glucose production

In the mRNA expression of IRB, SLC2A2, and PCK1A, an interaction of d × NA was found. For SLC2A2 and IRB, the relative quantities of mRNA at d1 was lower in NA groups compared with the CON groups (Table 2). Moreover, for SLC2A2 and PCK1, the relative quantities of mRNA were higher at d21 in NA groups compared with in CON groups. Similar up-regulation by NA at d21 was found for G6P, which was reflected in a trend effect of the d × NA interaction. Significant d × concentrate interaction effects were found in SLC2A2, IRB, and PYGL. These interaction effects were caused by the lower relative quantities of mRNA at d21 in HC groups compared with in LC groups for the affected genes SLC2A2, IRB and PLGL.

In the mRNA expression of IRA, G6P, PCCA and PC, only time had a significant effect. The relative quantities of mRNA of IRA and PC were up-regulated at d1 and down-regulated at d21 and d100 (d-42, d100 < d21 < d1). A continuous up-regulation was observed in G6P (d-42 < d1 < d21 < d100). The relative quantity of mRNA of PCCA was down-regulated at d1 and then up-regulated at d21 and d100 (d1 < d21, d-42 < d100).

### Correlation analysis

**Fig 3:**
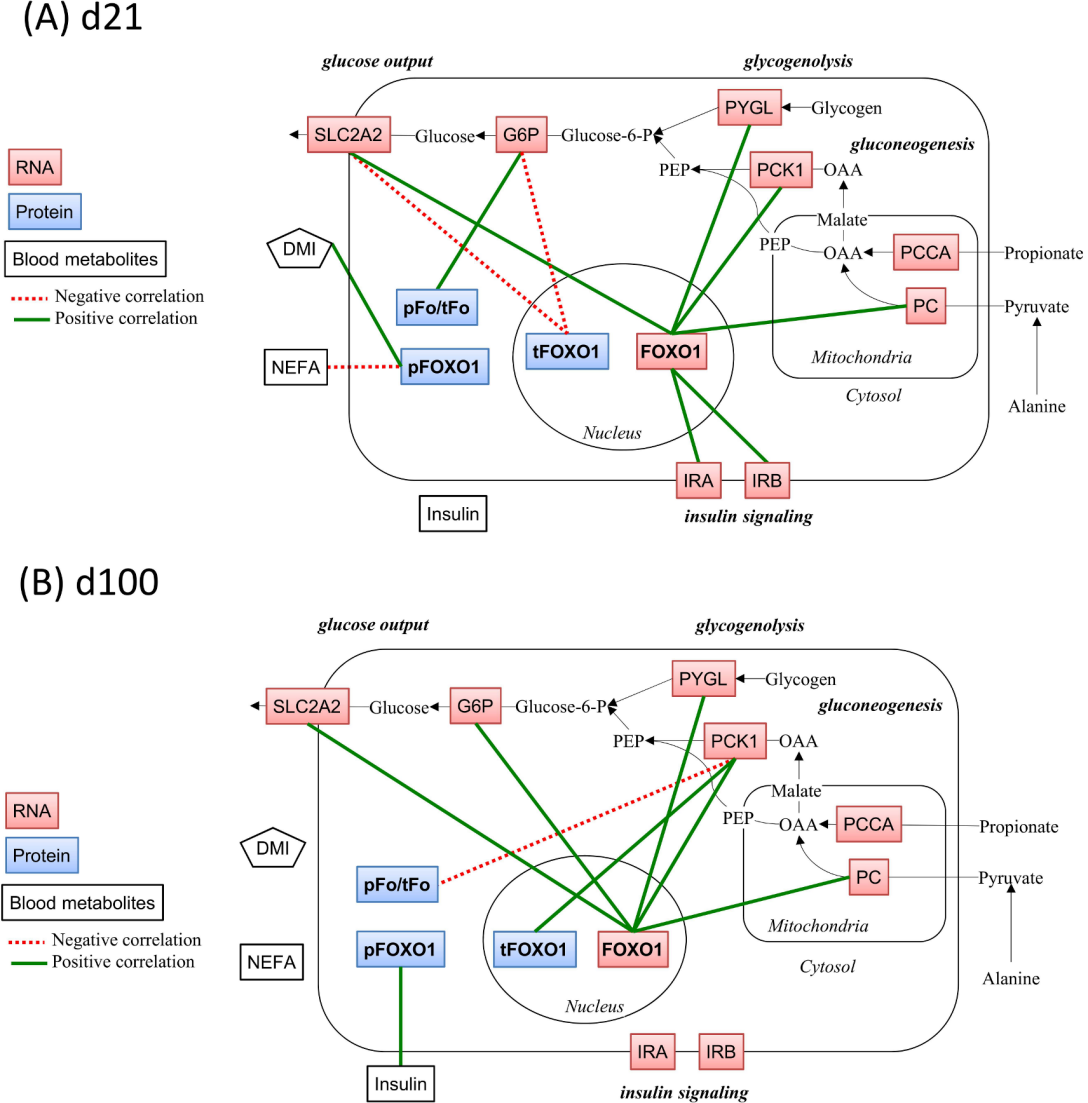
Correlations between FoxO1-related variables and other investigated variables in the hepatic metabolic pathways related to gluconeogenesis. To demonstrate correlations graphically, the scheme was adopted to one published by Aschenbach et al. [11]. Pearson’s correlation analyses were performed using the data set from d21 (A) and d100 (B) (N = 21 dairy cows for each). Variables with positive correlations were connected by green solid lines and those with negative correlations by red dotted lines. The level of significance was at least P ≤ 0.01. The metabolites connected by black arrows show the representative metabolic pathways related to gluconeogenesis (not investigated, OAA: Oxaloacetate, PEP: Phosphoenolpyruvate, Glucose-6-P: Glucose-6-phosphate). The variables other than DMI used in the correlation analysis are shown in boxes (blue, red, white for hepatic protein expression, hepatic mRNA expression, and blood metabolites, respectively). FoxO1: Forkhead box protein O1, tFoxO1: Total protein expression of FoxO1, pFoxO1: Extent of phosphorylation of FoxO1 at serine 256, pFo/tFo: pFoxO1-to-tFoxO1 ratio, PCCA: Propionyl CoA carboxylase A, PC: Pyruvate carboxylase, PCK1: Cytosolic phosphoenolpyruvate carboxykinase, G6P: Glucose-6-phosphatase, SLC2A2: Glucose transporter 2 (solute carrier family 2 (facilitated glucose transporter), member 2), IRA, IRB: Insulin receptor isoform A and B, PYGL: Glycogen phosphorylase, liver form, DMI: Dry matter intake, NEFA: Serum concentration of non-esterified fatty acid, Insulin: Insulin serum concentration.

No significant correlation was found at **d-42 and d1** (data not shown). At **d21 the pFoxO1** (protein amount) correlated positively with DMI (0.70, P < 0.001). The mRNA expression of **FoxO1** correlated positively with mRNA expression of SLC2A2 (0.6, P = 0.004), IRB (0.75, P < 0.001), PC (0.59, P = 0.005). At **d100** the **tFoxO1** (protein amount) correlated positively with mRNA expression of PCK1 (0.64, P = 0.002), **the pFoxO1** (protein amount) correlated positively with insulin (0.63, P = 0.004). The mRNA expression of **FoxO1** correlated positively with mRNA expression of SLC2A2 (0.63, P = 0.004), PCK1 (0.73, P < 0.001), G6P (0.61, P = 0.008), and PYGL (0.60, P = 0.01). The **ratio of pFoxO1/tFoxO1** (protein amount) correlated negatively with mRNA expression of PCK1 (-0.53, P = 0.01). Graphical demonstrations of the correlation at d21 and at d100 are presented in Fig 3.

## Discussion

The objective of this study was to assess the role of FoxO1 in HGP in dairy cows during the transition period. Therefore, we aimed to find indicative associations between FoxO1 expression, the extent of phosphorylation of FoxO1 at serine 256, serum insulin concentration and expression of mRNA of genes associated with HGP as affected by the feeding regimen. To reveal associations between FoxO1 and metabolic status, DMI and serum NEFA concentration were also included in the correlation analysis.

We were able to show that FoxO1 mRNA and protein expression were constant during the transition period and were not affected by diet. In the extent of phosphorylation of FoxO1, an interaction of NA × concentrate was found. However, no corresponding NA × concentrate interaction effect was found in serum insulin concentration and mRNA expression of genes involved in HGP. In the correlation analysis, different correlation patterns were observed at d21 and d100.

### FoxO1 expression and phosphorylation at serine 256

FoxO1 is a main mediator of effect of insulin to inhibit HGP in monogastric species [30,31]. FoxO1 has three Akt phosphorylation sites as targets of insulin signaling. Among three phosphorylation sites, the phosphorylation at serine 256 is needed for the subsequent phosphorylation of the other two sites so that it acts as a gatekeeper [32,33]. Liver-specific knockout of FoxO1 led to more than 50% down-regulation of the transcription of G6P and PCK1 in hyperglycemic mice. This down-regulation of G6P and PCK1 was accompanied by reducing blood glucose level in these mice [34,35]. As to effects of diet on FoxO1 expression, Grala et al. [5] observed ×1.2 up-regulation of FoxO1 mRNA together with up-regulations of mRNA of other gluconeogenic enzymes in the liver in dairy cows fed a restricted diet for three weeks in early lactation compared with control cows.

We observed lower insulin concentrations as well as an up-regulation of mRNA expression of several of the gluconeogenic enzymes after calving (discussed below). These results indicated an increase of HGP after calving. However, the glucose flux in the liver was not measured in this study. An up-regulation of FoxO1 and/or down-regulation of the pFoxO1 were expected. However, the amount of FoxO1 protein and mRNA, as well as that of pFoxO1 did not differ between the sampling days. This could suggest that FoxO1 expression and pFoxO1 most likely did not account for the up-regulation of gluconeogenic enzymes after calving. Similarly, Zachut et al. [36] observed an almost equal extent of the phosphorylation of hepatic insulin receptor and Akt by insulin before and after calving in high-yielding Holstein dairy cows. This confirms that phosphorylation of FoxO1 postpartum did not change in this study.

Regarding the effects of the diet, we assumed that NA promotes the HGP by up-regulating the FoxO1 as well as by inhibiting the phosphorylation of FoxO1 by insulin. Moreover, we assumed that there are interactions between concentrate intake before calving and NA supplementation. However, results of analysis in FoxO1 indicated that there appeared to be no direct relationship between the dietary effects found in FoxO1 analysis and those on the other variables. Interestingly, in rats, a NA infusion induced a significantly lower extent of phosphorylation of insulin receptor and Akt, but not FoxO1 at serine 256 in liver [19].

Our results could indicate that HGP was not regulated by FoxO1 on the levels of mRNA and protein expression, and of the phosphorylation at serine 256 in dairy cows during the transition period. This interpretation may support the fact that the HGP in dairy cows was insensitive to insulin [6,11]. However, it was also possible that the other posttranslational regulation mechanisms are of more importance in the FoxO1-mediated regulation on HGP [2]. For example, NA supplementation might increase NAD concentration and activate a NAD-dependent deacetylase (Sirtuin-1). The activated Sirtuin-1 could promote the deacetylation of FoxO1 and thereby increase the transcriptional activity of the FoxO1 [2]. Thus, further studies are needed on the function of FoxO1 in the regulation of HGP in dairy cows during the transition period.

### Serum insulin and hepatic mRNA expression

In the serum insulin concentration and hepatic mRNA expression of genes associated with glucose production, time- and diet- related changes were detected. The lower insulin levels shortly after calving [36,37] and the up-regulation of mRNA of PC, PCK1, and G6P [8–10,36,38] were in line with the results in other studies. These changes reflected the homeorhetic adaptation of cows [6]. The pattern of mRNA expression of PCCA was parallel to the changes of DMI [20,21], as also observed by Graber et al. [38]. As the PCCA is needed to catalyzes the conversion of propionate to succinyl-CoA [11], these time-related changes of PCCA most likely indicated the adaptation of hepatic tissues to an increased proportion of propionate as gluconeogenic precursor at d21. The increased expression of insulin receptors during the early lactation period was reported previously on both mRNA and protein levels, and was suggested to be an indicator for negative energy balance [36,39,40].

The mechanisms for the observed NA effects on hepatic mRNA expression are unknown. Based on studies in monogastric species, Kang et al. [41] proposed that NA could modify the gene expression by interacting with NA receptor, by changing the profiles of blood metabolites, and by activating FoxO1. In the current study, the NA effect found on the mRNA expression could not be completely explained by the regulation of FoxO1 or by blood metabolites. It might be possible that a part of the NA-induced modification of mRNA expression in the current study occurred through the activating NA receptors, because bovine liver expressed a substantial amount of NA receptor [17].

### Correlation analysis

Correlations at each sampling day could be interpreted as coordinated changes of two variables for the short-time metabolic adaptation. The fact that the correlation pattern differed among the sampling days was previously observed [42]. This result indicated the shift of regulation mechanisms for HGP during the transition period.

Lack of any correlations between FoxO1 and other variables at d-42 and d1 confirmed that the FoxO1 was not involved in the regulation of HGP during this period. In spite of lack of any significant effects on rFoxO1, it correlated positively with the mRNA expressions of the other genes, which were significantly affected by time and diet. This was observed both at d21 and d100, and could reflect the multilevel regulation mechanism of transcriptional activity of FoxO1. The proportional impact of regulation of the FoxO1 activity at the level of mRNA was probably too small to be detected by factorial analysis. The positive correlation for pFoxO1–DMI found at d21 could indicate that the FoxO1 in inactive form decreased in cows with energy deficit. However, lack of any other correlations for tFoxO1 and pFoxO1 at d21 could indicate that FoxO1-related regulation was not dominant in the regulation of HGP at d21. In contrast, the positive correlation for PCK1−tFoxO1 and, although the correlation coefficient was relatively weak, the negative correlation for PCK1− ratio of pFoxO1/tFoxO1 at d100 were in accordance with the hypothesis. These could indicate that PCK1 was up-regulated by FoxO1 protein, and that this up-regulation was inhibited by insulin-induced phosphorylation of FoxO1.

Altogether, the results in the correlation analysis could suggest that the HGP could be regulated by FoxO1 and insulin in dairy cows only in the mid lactation, and not in the period of late pregnancy and early lactation.

## Conclusion

The results indicated that the main regulation of HGP did not take place on the levels of mRNA and protein expression of FoxO1 or on the phosphorylation of FoxO1 at serine 256 in dairy cows from d-42 to d21. However, at d100 FoxO1 appeared to be responsible for at least a part of the regulation mechanism of HGP. The less dominance of FoxO1 as a mediator of insulin-effect on HGP could support the insensitivity of HGP to the suppressive effect of insulin in dairy cows. However, the possibility was not ruled out that the posttranslational modification of FoxO1 plays a more important role in the regulation of HGP. Therefore, further study is needed to clarify the roles of FoxO1 in the regulation of HGP in dairy cows during the transition period.

## Supporting Information

S1 Fig Signals of total protein of FoxO1 (a), phosphorylated FoxO1 at serine 256 (b) and negative control (c) in Western Blot analysis. Thirty μg of reduced and denatured proteins isolated from liver were transferred to nitrocellulose membrane. Membrane was blocked with 10% skimmed milk-TBST for 1h at room temperature and incubated with primary antibodies diluted at 1:1000 (a) or 1:200 (b) in 5% skimmed milk-TBST or in 5% skimmed milk without primary antibody (c) over night at 4°C, followed by incubation with secondary antibodies diluted at 1:20000 5% skimmed milk-TBST at room temperature for 1h. Arrows indicate the molecular weight of 70 kD which positive signals are supposed to have (a, b). The signals appearing at 100 kD in a and c were considered to be unspecific as indicated by non-primary antibody control (c).
(TIFF)

S2 Fig Representative signals of total protein of FoxO1 and phosphorylated FoxO1 at serine 256 (70 kD) of cows from each experimental group and sampling time. Thirty μg of reduced and denatured proteins isolated from liver were transferred to nitrocellulose membrane. Membrane was blocked with 10% skimmed milk-TBST for 1h at room temperature and incubated with primary antibodies diluted at 1:1000 (tFoxO1) or 1:200 (pFoxO1) in 5% skimmed milk-TBST over night at 4°C, followed by incubation with secondary antibodies diluted at 1:20000 5% skimmed milk-TBST at room temperature for 1h. Signals of beta-actin (45 kD) are presented as internal controls. tFoxO1: total protein of FoxO1, pFoxO1: phosphorylated FoxO1 at serine 256, LC-CON, HC-CON, LC-NA, HC-NA: “CON or NA”: Nicotinic acid (0 or 24 g/d) from d-42 to d24, “LC or HC”: 30 or 60% of concentrate proportion in the diet from d-42 to d0, increase of concentrate proportion in the diet after calving from 30 to 50% within 16 or 24 days, control: A control sample for inter membrane controls, d: Days related to calving.
(TIFF)

S3 Fig Agarose gel electrophoresis of PCR products. About 7 μl of PCR products were applied to 2% agarose gel with Tris-acetate-EDTA-buffer, run at 75 V for 50 min. The gel was stained with SYBR Green for 95 min. 1: Marker, 2: MRPL39, 3:RPS15, 4: RBMS2, 5: UXT, 6: RPS9, 7: RPL32, 8: RPL19, 9: Marker; 10: PC, 11: PCCA, 12: PCK1, 13: G6P, 14: SLC2A2, 15: CPT1 (172 bp, not measured in this study), 16: PYGL, 17: IRB, 18: IRA, 19: FoxO1, 20: Empty.
(TIFF)

S1 Table Results of the sequencing of PCR products and BLAST analysis^1^. FoxO1: Forkhead box protein O1, IRA: Insulin receptor isotype A, IRB: Insulin receptor isotype B, SLC2A2: Glucose transporter 2 (Solute Carrier Family 2 (Facilitated Glucose Transporter), Member 2), G6P: Glucose-6-phosphatase, PCK1: Cytosolic phosphoenolpyruvate carboxykinase, PCCA: Propionyl-CoA carboxylase, PYGL: Glycogen phosphorylase, RPL19: Ribosomal protein L19: Ribosomal protein L32, RPS9: Ribosomal protein S9, UXT: Ubiquitously expressed transcript, MRPS15: Mitochondrial ribosomal protein S15, RBMS2: RNA binding motif, single strand interacting protein 2, MRPL39: Mitochondrial ribosomal protein L39. ^1^ Analysis performed using Basic Local Alignment Search Tool “BLASTN 2.2.31+”, an online program offered by National Center for Biotechnology Information (Bethesda, USA) [24] and the database Bos taurus Annotation Release 104 RNAs.
(PDF)
