# Supplementary figures and images for: Associations between Forkhead Box O1 (FoxO1) Expression and Indicators of Hepatic Glucose Production in Transition Dairy Cows Supplemented with Dietary Nicotinic Acid
Source: PLoS One. 2016 Jan 22;11(1):e0146670. doi: 10.1371/journal.pone.0146670 (PMC4723333; doi:10.1371/journal.pone.0146670)

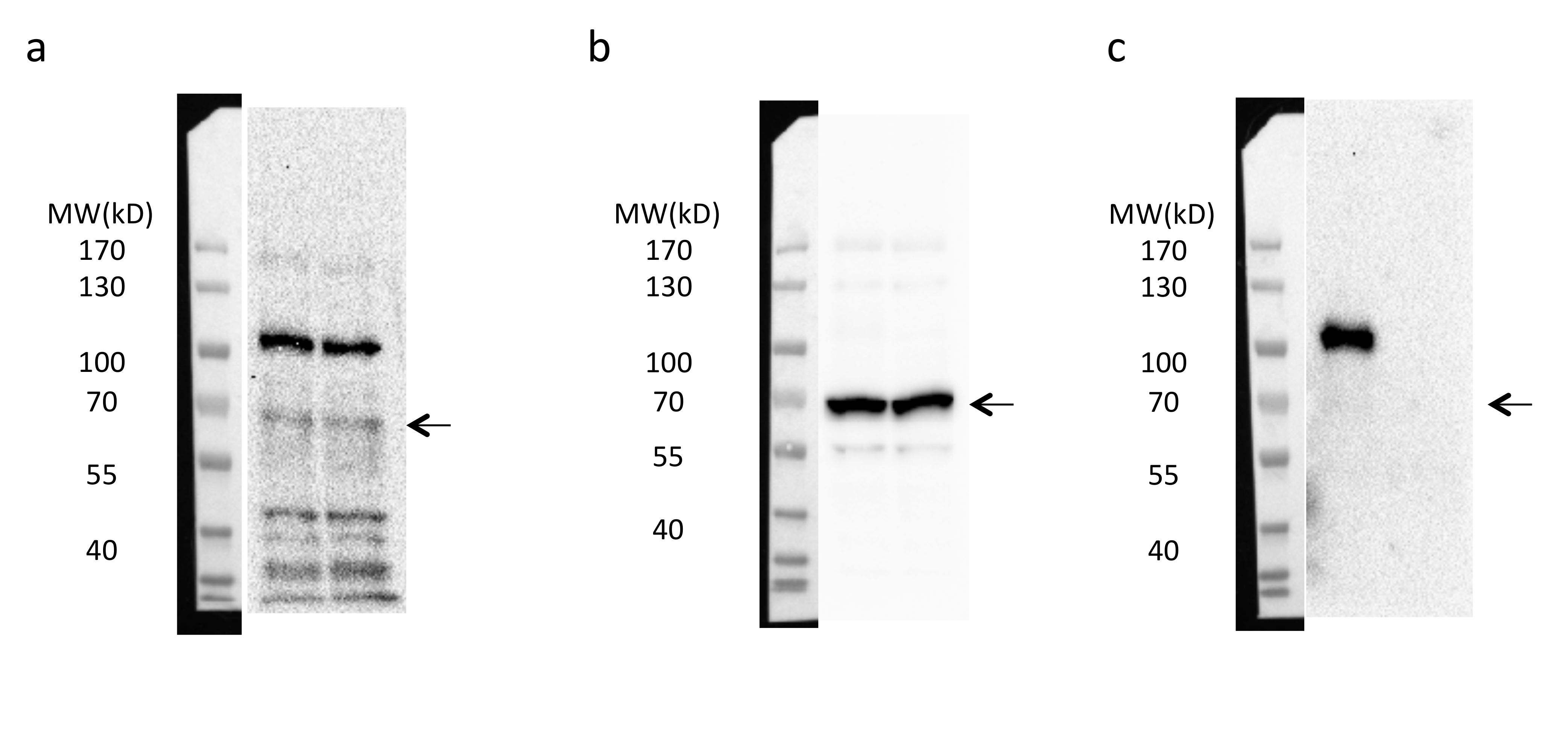

Supplement: S1 Fig — Thirty μg of reduced and denatured proteins isolated from liver were transferred to nitrocellulose membrane. Membrane was blocked with 10% skimmed milk-TBST for 1h at room temperature and incubated with primary antibodies diluted at 1:1000 (a) or 1:200 (b) in 5% skimmed milk-TBST or in 5% skimmed milk without primary antibody (c) over night at 4°C, followed by incubation with secondary antibodies diluted at 1:20000 5% skimmed milk-TBST at room temperature for 1h. Arrows indicate the molecular weight of 70 kD which positive signals are supposed to have (a, b). The signals appearing at 100 kD in a and c were considered to be unspecific as indicated by non-primary antibody control (c). (TIFF) [file pone.0146670.s001.tiff]

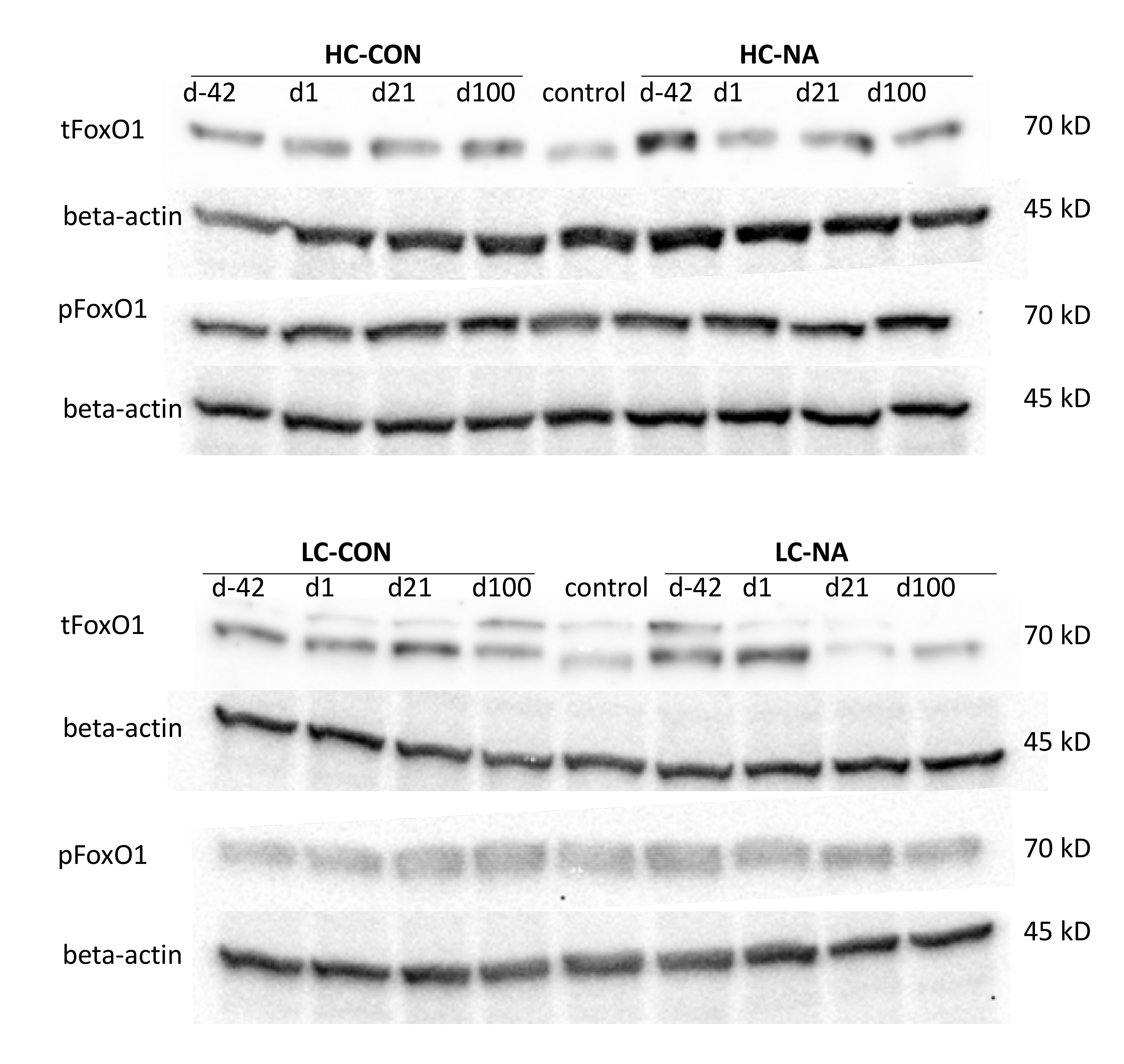

Supplement: S2 Fig — Thirty μg of reduced and denatured proteins isolated from liver were transferred to nitrocellulose membrane. Membrane was blocked with 10% skimmed milk-TBST for 1h at room temperature and incubated with primary antibodies diluted at 1:1000 (tFoxO1) or 1:200 (pFoxO1) in 5% skimmed milk-TBST over night at 4°C, followed by incubation with secondary antibodies diluted at 1:20000 5% skimmed milk-TBST at room temperature for 1h. Signals of beta-actin (45 kD) are presented as internal controls. tFoxO1: total protein of FoxO1, pFoxO1: phosphorylated FoxO1 at serine 256, LC-CON, HC-CON, LC-NA, HC-NA: “CON or NA”: Nicotinic acid (0 or 24 g/d) from d-42 to d24, “LC or HC”: 30 or 60% of concentrate proportion in the diet from d-42 to d0, increase of concentrate proportion in the diet after calving from 30 to 50% within 16 or 24 days, control: A control sample for inter membrane controls, d: Days related to calving. (TIFF) [file pone.0146670.s002.tiff]

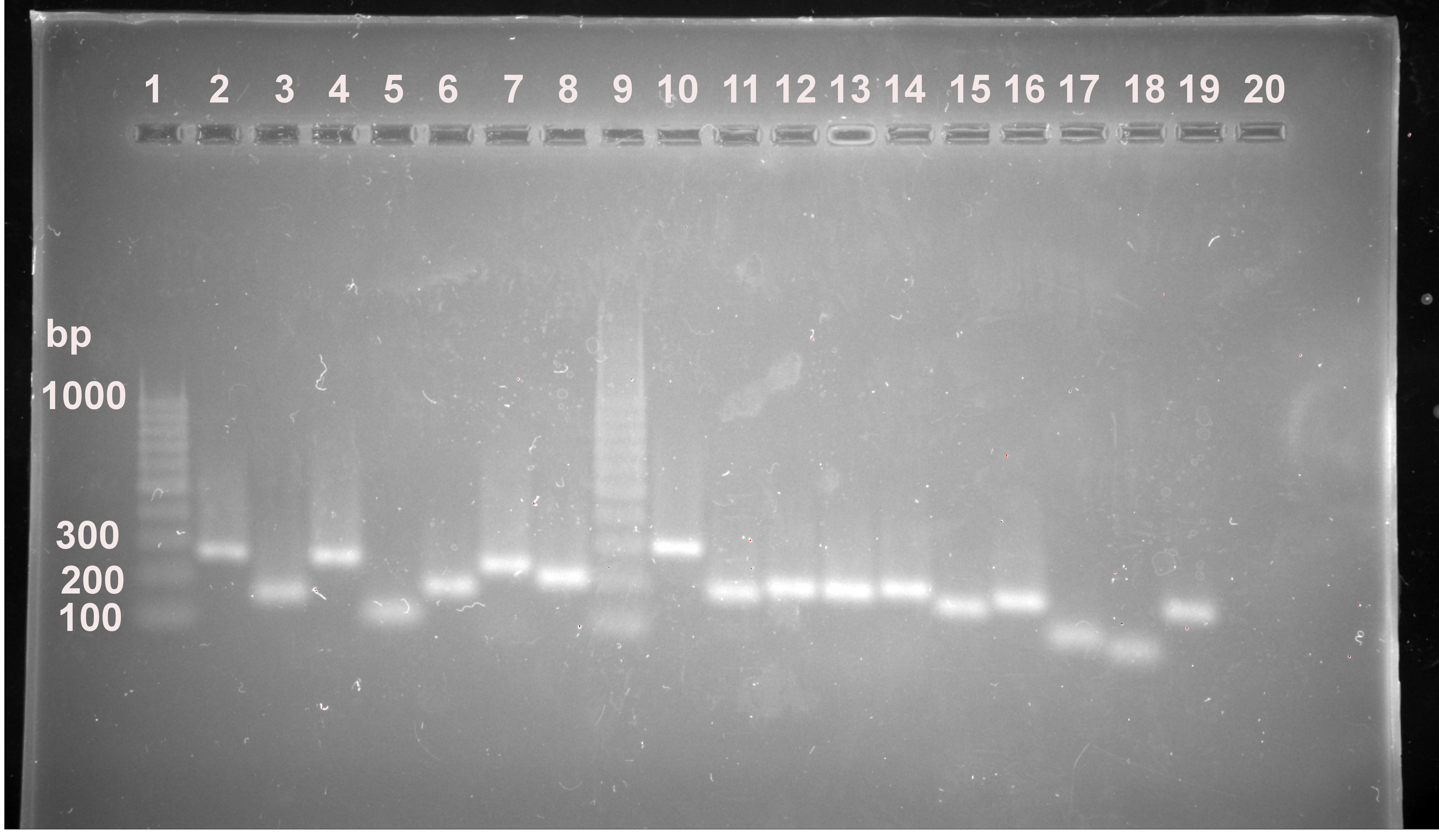

Supplement: S3 Fig — About 7 μl of PCR products were applied to 2% agarose gel with Tris-acetate-EDTA-buffer, run at 75 V for 50 min. The gel was stained with SYBR Green for 95 min. 1: Marker, 2: MRPL39, 3:RPS15, 4: RBMS2, 5: UXT, 6: RPS9, 7: RPL32, 8: RPL19, 9: Marker; 10: PC, 11: PCCA, 12: PCK1, 13: G6P, 14: SLC2A2, 15: CPT1 (172 bp, not measured in this study), 16: PYGL, 17: IRB, 18: IRA, 19: FoxO1, 20: Empty. (TIFF) [file pone.0146670.s003.tiff]
