# Supplementary material for: Associations between Forkhead Box O1 (FoxO1) Expression and Indicators of Hepatic Glucose Production in Transition Dairy Cows Supplemented with Dietary Nicotinic Acid
Source: PLoS One. 2016 Jan 22;11(1):e0146670. doi: 10.1371/journal.pone.0146670 (PMC4723333; doi:10.1371/journal.pone.0146670)
Supplement: S1 Table — FoxO1: Forkhead box protein O1, IRA: Insulin receptor isotype A, IRB: Insulin receptor isotype B, SLC2A2: Glucose transporter 2 (Solute Carrier Family 2 (Facilitated Glucose Transporter), Member 2), G6P: Glucose-6-phosphatase, PCK1: Cytosolic phosphoenolpyruvate carboxykinase, PCCA: Propionyl-CoA carboxylase, PYGL: Glycogen phosphorylase, RPL19: Ribosomal protein L19: Ribosomal protein L32, RPS9: Ribosomal protein S9, UXT: Ubiquitously expressed transcript, MRPS15: Mitochondrial ribosomal protein S15, RBMS2: RNA binding motif, single strand interacting protein 2, MRPL39: Mitochondrial ribosomal protein L39. 1 Analysis performed using Basic Local Alignment Search Tool “BLASTN 2.2.31+”, an online program offered by National Center for Biotechnology Information (Bethesda, USA) [24] and the database Bos taurus Annotation Release 104 RNAs. (PDF) [file pone.0146670.s004.pdf]

S4 Table. Results of the sequencing of PCR products and BLAST analysis[1].

Symbol	Sequence results of PCR products	BLAST hit gene (NCBI) description	Identities
FoxO1	CATTCTGCACGCGGATGAACTTGCTGTGCAGGGACAGATTATGACGAATTGA<br>ATTCTTCCAGCCCGCTGAGCTGTTGCTGTCGCCCTTATCCTTGAAGTAGGGCA<br>CGCTCTTGACCATCCACTCGTAAATCTGCG	PREDICTED: Bos taurus forkhead box O1 (FOXO1), transcript variant X2, mRNA	135/135
G6P	AGCAGGTGTATACTATGTGATGGTCACATCCACCCTCTCTATCTTTCGTGGAA<br>AGAAAAAGCCAACCTACAGATTTCGGTGCTTGAATGTCATGTTGTGGTTGGG<br>ATTCTGGGTCGTGCAACTGAA	Bos taurus glucose-6-phosphatase, catalytic subunit (G6PC), mRNA	126/126
SLC2A2	GAGAGGCATATCAGGACTCTACTGTGGGCTGATTTCAGGCTTGATTCCAATG<br>TACATTGGTGAAATTGCTCCAACCACACTCAGGGGCGCTATCGGTGCTCTTC<br>ATCAGCTGGCCATTGTCACGGGCATTCTTATTAGTCAGATCGTTGGCCTTGAC<br>TTTATCCTGGGCA	Bos taurus solute carrier family 2 (facilitated glucose transporter), member 2 (SLC2A2), mRNA	169/169
IRA	AAGATTACCTGCACAACGTGGTTTTCATCCCCAGGCCTTCAAGGAGCTGGAG<br>GAGTTGAT	PREDICTED: Bos taurus insulin receptor (INSR), transcript variant X8, mRNA	44/45
IRB	GTTCTTCAAGGAGCTGGAGGAGTCCTCGTTCAGGAAAACGTTTGAAGATACC<br>TGCACAACGTGGTTCTCATCCCCAGAAAATCATCTTCAGGCCCTGTTGCCGA<br>GGACGCTAAATT	Bos taurus insulin receptor (INSR), transcript variant X6, mRNA	106/108
PC	CAGCTGCGCACCCGGCTCACTAGCGACTCCGTCAAGCTTGCAAAGCAGGTGG<br>GCTACGAGAACGCGGGCACCGTGGAGTTCCTGGTGGACAGGCACGGCAAGC<br>ACTACTTCATCGAGGTCAACTCGCGCCTGCAGGTGGAGCACACGGTGACCGA<br>GGAGATCACAGATGTGGACCTGGTCCACGCCCAGATCCACGTGGCCGAGGG<br>CCGGAGCCTGCCTGACCTGGGCCTCCGGCAGGAGAACATCCGCATCAACCAG<br>CT	Bos taurus pyruvate carboxylase, mRNA	254/255
PCCA	TGGGTCGACTAAATGTGACCAGCACGTGGAACCTGGCTTCACCCTTGTTGTC<br>TGTCAACGTTGATGGCACTCAGAGGACGATACAGTGTCTTTCTCGAGAAGCA<br>GGGGGCAACATGAGCATTCAGTTTCTCGGCACAGTAA	Bos taurus propionyl CoA carboxylase, alpha polypeptide (PCCA), mRNA	139/141
PCK1	AGGACGGCAGCTGCTGAGCCACATGGAGGAGGAGGGTGTGATCAAGAGGCT<br>GAAGAAGTATGACAACTGCTGGTTGGCTCTCACTGACCCCAGGGATGTGGCC<br>AGAATTGAAAGCAAGACGGTCATCATCACTCGAGAGCAAAGAGATACGGTG<br>CCCATCAATT	Bos taurus phosphoenolpyruvate carboxykinase 1 (soluble) (PCK1), mRNA	156/156
PYGL	AGTTCTGGAGAGGAGTACAAAGTGAAGATCAACCCGTCCTCCATGTTCGACG<br>TGCAGGTGAAGCGGATCCACGAGTACAAGCGACAGCTCCTGAACTGCCTGC<br>ACGTGGTCACCATGTACAACCGCATTAAGAAAGACCCAAAGAAG	Bos taurus phosphorylase, glycogen, liver (PYGL), mRNA	146/149

S4 Table. (continued)

Symbol	Sequence results of PCR products	BLAST hit gene (NCBI) description	Identities
RPL19	AATTCTGCGCCGGCTGCTTAGACGATACCGTGAATCTAAGAAGATTGACCGCCACATGTATCACAGCCTGTACCTGAAGGTGAAGGGTAACGTGTTCAAAAACAAGCGGATCCTCATGGAACATATCCACAAGCTGAAGGCAGACAAAGTGGTCGGC	Bos taurus ribosomal protein L19 (RPL19), mRNA	147/147
RPL32	TCATTAGGCATCAATCAGACCGATATGTCAAAATCAAGCGGAACTGGCGGAAGCCCAGAGGCATTGACAACAGGGTGCGCAGAAGATTCAAGGGCCAGATCTTGATGCCCAACATTGGTTATGGGAGCAACAAGAAAACCAAGCACATGCTGCCCAGCGGCTTCCGGAAGTTCCTGGTCCACAACGTCAAACTCTTC	Bos taurus ribosomal protein L32 (RPL32), mRNA	190/190
RBMS2	CAGCAGTGGGAGCACTGCACATGACCAGCTGAGTAAGACCAACCTCTACATCCGGGGATTGCAGCCAAGCACTACTGACCAGGACCTAGTGAAGTTGTGTCAGTCATACGGCAAGATTGTCTCCACTAAGGCCATCCTGGACAAGACCACAAACAAGTGCAAAGGCTATGGCTTTGTGGACTTCGACAGTCCTTCAGCAGCACAGAAAGCAGTGAATTT	Bos taurus RNA binding motif, single stranded interacting protein 2 (RBMS2), mRNA	213/213
MRPL39	ACTGACCCTGGCACTATCTTTGTGTTGAATAAAAACGTTTCCACTCCTTATAGCTGTGCCATGCATTTAAGTGAGTGGTACTGCAGGAAGTCTATTCTGGCTCTTGTGGACGGACAGCCTTGGGACATGTATAAACCTTTGACCAAGTCCTGTGAAATTAAATTTCTAACTTTCAAAGATGATGATCCAGGAGAAGTCAATAAGGCTTATTGGCGTTCTTGTGCCCATATT	Bos taurus mitochondrial ribosomal protein L39 (MRPL39), mRNA	225/225
UXT	GGCAGAGCTCTCAGTTCATTGATCGTAAGAGCAGTCTCCTCACAGAGCTCAGCGACAACATGG	Bos taurus ubiquitously-expressed transcript (UXT), mRNA	59/61
MRPS15	CCTGGAGGCTCAATTGTTGCCTTGACTGTCAAGATCCGCAGTTACGAAGAACACATGCAGAAACATCAAAAGGACAAAGCCCACAAGCGCTATCTGCTGATGAGCAAGTTG	Bos taurus mitochondrial ribosomal protein S15 (MRPS15), mRNA	106/107
RPS9	GGCTGCCGGGAGCTGCTGACGCTGGATGAGAAAGACCCGCGGCGTCTGTTCGAAGGTAATGCCCTGTTGCGGCGGCTCGTCCGTATCGGGGTGCTGGATGAGGGCAAGATGAAGCTGGATTACATCCTGGGAAGGGCTTC	Bos taurus ribosomal protein S9 (RPS9), mRNA	131/132
